# Real-Time Control of a Neuroprosthetic Hand by Magnetoencephalographic Signals from Paralysed Patients

**DOI:** 10.1038/srep21781

**Published:** 2016-02-24

**Authors:** Ryohei Fukuma, Takufumi Yanagisawa, Youichi Saitoh, Koichi Hosomi, Haruhiko Kishima, Takeshi Shimizu, Hisato Sugata, Hiroshi Yokoi, Masayuki Hirata, Yukiyasu Kamitani, Toshiki Yoshimine

**Affiliations:** 1 Osaka University Graduate School of Medicine, Department of Neurosurgery, Suita 565-0871, Japan; 2 ATR Computational Neuroscience Laboratories, Department of Neuroinformatics, Seika-cho 619-0288, Japan; 3 Nara Institute of Science and Technology, Graduate School of Information Science, Ikoma 630-0192, Japan; 4 Osaka University Graduate School of Medicine, Division of Functional Diagnostic Science, Suita 565-0871, Japan; 5 Osaka University Graduate School of Medicine, Department of Neuromodulation and Neurosurgery, Suita 565-0871, Japan; 6 The University of Electro-Communications, Department of Mechanical Engineering and Intelligent Systems, Chofu 182-8585, Japan; 7 Kyoto University, Graduate School of Informatics, Kyoto 606-8501, Japan

## Abstract

Neuroprosthetic arms might potentially restore motor functions for severely paralysed patients. Invasive measurements of cortical currents using electrocorticography have been widely used for neuroprosthetic control. Moreover, magnetoencephalography (MEG) exhibits characteristic brain signals similar to those of invasively measured signals. However, it remains unclear whether non-invasively measured signals convey enough motor information to control a neuroprosthetic hand, especially for severely paralysed patients whose sensorimotor cortex might be reorganized. We tested an MEG-based neuroprosthetic system to evaluate the accuracy of using cortical currents in the sensorimotor cortex of severely paralysed patients to control a prosthetic hand. The patients attempted to grasp with or open their paralysed hand while the slow components of MEG signals (slow movement fields; SMFs) were recorded. Even without actual movements, the SMFs of all patients indicated characteristic spatiotemporal patterns similar to actual movements, and the SMFs were successfully used to control a neuroprosthetic hand in a closed-loop condition. These results demonstrate that the slow components of MEG signals carry sufficient information to classify movement types. Successful control by paralysed patients suggests the feasibility of using an MEG-based neuroprosthetic hand to predict a patient’s ability to control an invasive neuroprosthesis via the same signal sources as the non-invasive method.

Severely paralysed patients and amputees can activate their sensorimotor cortices by attempting to move their affected limb^1^. Invasively recorded signals, such as those taken from an electrocorticogram (ECoG), showed that a patient’s attempt to move his paralysed arm resulted in modulations in cortical potentials in the sensorimotor cortex that were substantial enough to control a prosthetic arm^2^. Interestingly, the characteristic features of the potentials needed to control the neuroprosthesis were similar to those of non-paralysed subjects: slow cortical potentials (SCPs) and powers of the alpha (8–13 Hz), beta (13–30 Hz), and high-γ (80–150 Hz) frequency ranges^3^,^4^,^5^,^6^. Moreover, these features are also observed in non-invasively recorded signals such as magnetoencephalography (MEG) and electroencephalography^7^. Notably, even when taken from non-invasive signals, these features can infer the intention^8^,^9^,^10^, types^7^,^11^, and even trajectory^12^,^13^ of upper limb movements of both paralysed patients and non-paralysed subjects. In fact, we have demonstrated that the slow components of MEG signals (SMFs), can infer the type and intention of actual hand movements of healthy subjects accurately enough to control a prosthetic arm in real-time^14^. However, it has not yet been specifically determined whether the SMF can infer the attempted movements of paralysed patients precisely enough to control the neuroprosthesis, because the cortical potentials of the patients might be altered due to the paralysis.

Paralysis might affect the cortical potentials recorded over the motor cortex and, thus, the performance of a brain machine interface (BMI). Previous studies revealed that cortical reorganization took place in both hemispheres in accordance with the degree of motor dysfunction^15^,^16^,^17^,^18^,^19^,^20^,^21^,^22^,^23^,^24^,^25^,^26^, and that cortical potentials of paralysed patients were affected in amplitude and latency from the onset of movement^27^. Moreover, it has been shown that the ECoG of paralysed patients deteriorates in response to different movements, resulting in decreased accuracy of decoding^6^. Thus, the cortical potentials of severely paralysed patients should be non-invasively evaluated before the invasive treatment to predict the performance of neuroprostheses. In particular, the information represented by the SCP and frequency features should be estimated over the whole brain to evaluate the effect of cortical reorganization after paralysis.

In this study, we applied an MEG-based neuroprosthesis to eight severely paralysed patients suffering from brachial plexus root avulsion and to one amputee (Table 1), to evaluate the cortical currents representing the attempted movements of their affected hands and the performances in controlling the neuroprosthesis. MEG signals were recorded during attempts to grasp with or open their affected hand, and during actual execution by their intact hand (open-loop session, Fig. 1a). The obtained signals were converted into SMFs and some frequency powers to elucidate the appropriate features for controlling the prosthetic hand and a signal source reconstruction technique (variational Bayesian multimodal encephalography; VBMEG)^28^ was used to reveal the activated brain areas. Moreover, five patients underwent another measurement with real-time MEG to control a neuroprosthetic hand under a closed-loop condition using SMFs evoked by the attempted movements of their affected hand (Fig. 1b).

## Results

### Movement-Related Activity during the Open-Loop Session

The characteristic activation of MEG signals was observed similarly when subjects attempted to move their affected hands and when they actually moved their intact hands in the open-loop session. Figure 2a shows a representative mean contour map of the SMF, consisting of 500-ms, time-averaged and normalized MEG signals for each sensor (see Methods), at the time of execution cues for movement. For movements of both affected and intact hands, the map shows a dipole pattern centred at the parietal region. Moreover, the SMFs during attempted movements gradually increased around the execution cue, peaking during the attempted movements, and their amplitudes depended on the movement type (Fig. 2b, lower panel). These spatiotemporal properties of the SMFs represent characteristic features of the movement-related cortical field (MRCF)^29^ during actual movement (Fig. 2c, lower panel), and were similarly observed in all subjects for both affected and intact hands. The time-frequency analysis also showed that the temporal frequency pattern during attempted movement was similar to that during the actual hand movement, which is represented by event-related desynchronisation of the alpha and beta frequency range (Fig. 2b,c, upper panels).

To determine the signal source of the SMF, the cortical currents were estimated by VBMEG and time-averaged to obtain an estimated SCP (eSCP) in the same way as the SMF. As shown in Fig. 3a, when a patient attempted to move his completely paralysed right hand, eSCPs were clearly activated in the left sensorimotor cortex (contralateral to the tested hand) and depended on the movement type, similar to the activation during movement of his intact left hand (Fig. 3b). The differences in the eSCPs between the two types of movements were evaluated by one-way analysis of variance (ANOVA). The *F*-values, colour-coded on the reconstructed surface of the normalized brain, also show that the eSCP in the contralateral sensorimotor cortex varied significantly between the two movement types, similar to those during the actual hand movements (Fig. 3c). Notably, a significant *F*-value in the contralateral sensorimotor cortex was observed in all subjects for movements of their intact hand, and in eight of nine subjects (except subject 9) for attempted movements of their affected hands. These results suggest that, even during attempted movements of affected hands, the motor representations by cortical currents are preserved in the contralateral sensorimotor cortex, and are similar to those during actual movements of intact hands.

### Movement Decoding

**Figure 1:**
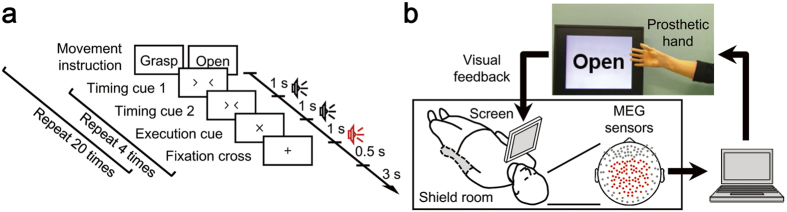
Experimental paradigm and system overview. (**a**) Experimental paradigm of the open-loop session. To begin, one of the movement types, grasp or open, was presented on the screen in front of the patient, followed by two “timing cues” and an “execution cue” at an interval of 1 s. The patient then attempted to move the affected hand as instructed at the timing of the “execution cue.” Each movement type was repeated four times. (**b**) System overview of the real-time prosthetic hand control. MEG signals from 84 parietal sensors, denoted by red dots, were acquired in real-time and analysed on a single computer. The prosthetic hand was controlled according to decoders that inferred the timing of movement intention and types of performed movements. The patient controlled the prosthetic hand by watching the screen representing the prosthetic hand and following the instructions for movements.

**Figure 2:**
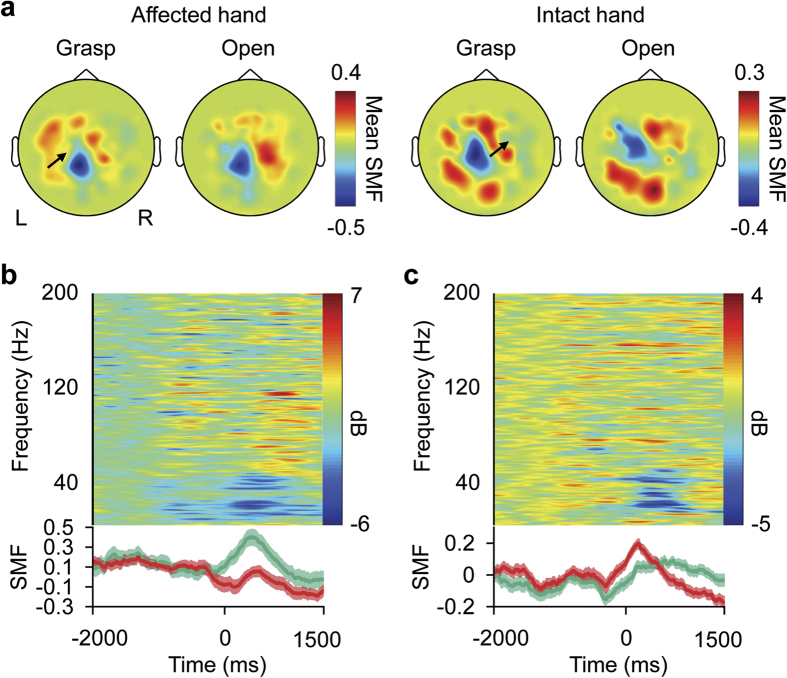
Measured magnetic fields. (**a**) Normalized slow components of the MEG signals (SMF) of subject 1 are shown during the attempts to use the completely paralysed (affected) right hand and during actual movements of the intact left hand. The SMFs were acquired from 0 to 500 ms relative to the execution cue for each sensor and colour-coded according to the colour bar at the location of each sensor. Black arrows indicate the sensors used in plots (**b**,**c**). R, right; L, left. (**b,c**) Upper panels show averaged power spectra of the MEG signals recorded during attempted hand grasping by the paralysed hand (**b**) or actual hand grasping by the intact hand (**c**) of subject 1; lower panels show the averaged SMFs during grasping and opening with green and red lines, respectively, and their respective standard errors as shaded areas. Time 0 ms denotes the execution cue time.

**Figure 3:**
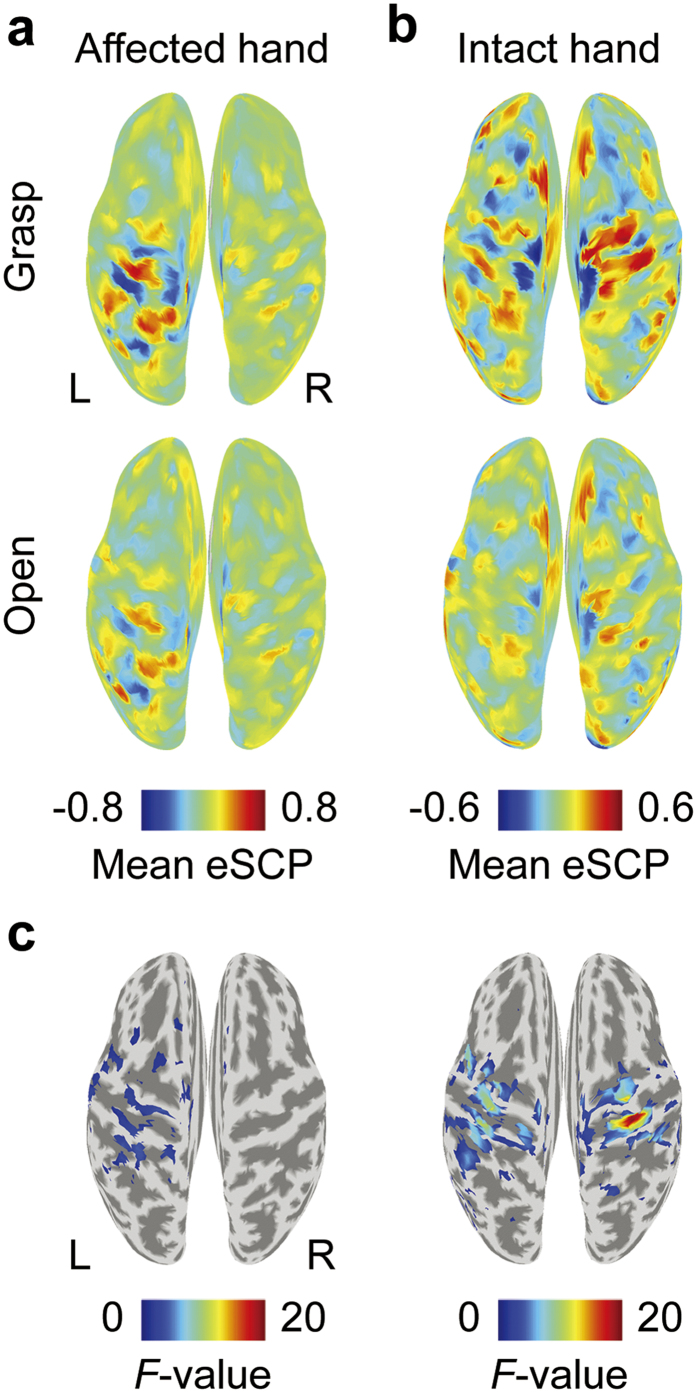
Measured cortical activity. (**a,b**) Normalized, estimated slow cortical potentials (eSCP) are colour-coded on the normalized brain surface during the attempts of subject 1 to grasp with or open his paralysed right hand (**a**) or performing the same movement with his intact hand (**b**). The eSCPs were acquired from 0 to 500 ms relative to the execution cue. R, right; L, left. (**c**) The one-way ANOVA *F-*values for the two movements shown in plots (**a,b**) are colour-coded on the normalized brain surface only for values with significant differences of *p* < 0.05.

The motor information about movement type and movement intention was evaluated by decoding analysis. Classification accuracies were compared between SMFs and powers of the alpha, beta, and high-γ bands. Movement type and intention features were extracted from the 84 parietal MEG sensors (Fig. 1b) and the analysis was performed using a nested cross-validation technique^30^ and a support vector machine (SVM, see Methods).

**Figure 4:**
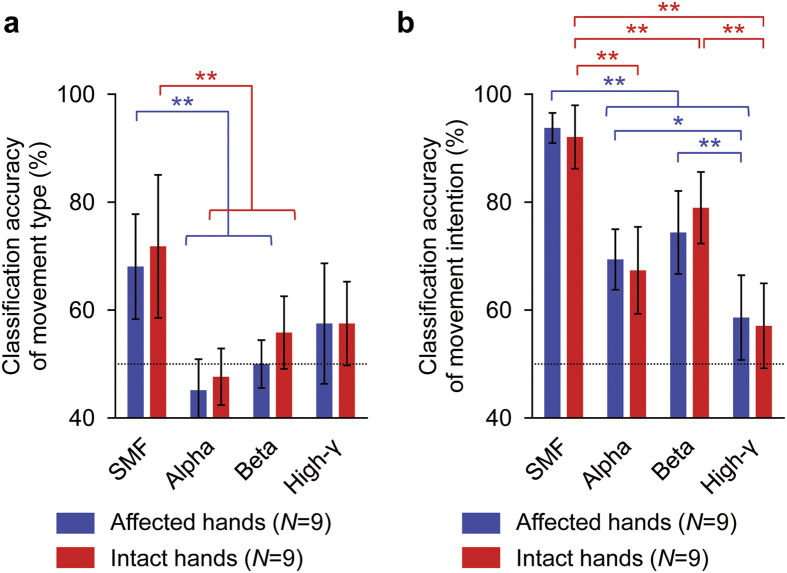
Classification accuracies for different features. (**a,b**) Blue and red bars show averaged classification accuracy of movement type (**a**) or movement intention (**b**) for affected and intact hands, respectively. Error bars show 95% confidence intervals of classification accuracy. Dotted lines denote chance level. *p < 0.05 and **p < 0.01 significant difference among tested hands (one-way ANOVA with post-hoc Tukey-Kramer).

Movement types were accurately classified using SMFs for both the affected and intact hands without significant difference (affected hand, 68.1 ± 12.7% (mean ± SD); intact hand, 71.8 ± 17.2%; paired two-tailed Student’s *t*-test, *p* = 0.5680; Fig. 4a). Notably, the classification accuracies varied significantly among different features for both affected and intact hands (affected hands: *p* = 0.0005, *F*(3,32) = 7.79, one-way ANOVA; intact hands: *p* = 0.0010, *F*(3,32) = 6.95). The classification accuracies using SMF were the highest among the tested features (affected hands: SMF vs. alpha band power, *p* = 0.0004, SMF vs. beta band power, *p* = 0.0060, SMF vs. high-gamma band power, *p* = 0.1790, post-hoc Tukey-Kramer test; intact hands: SMF vs. alpha band power, *p* = 0.0005, SMF vs. beta band power, *p* = 0.0277, SMF vs. high-gamma band power, *p* = 0.0564).

Similarly, movement intentions were accurately classified using SMFs for both the affected and intact hands without significant difference (affected hand, 93.8 ± 3.6% (mean ± SD); intact hand, 92.1 ± 7.6%; paired two-tailed Student’s *t*-test, *p* = 0.4407; Fig. 4b). The classification accuracies varied significantly among the decoding features (affected hands: *p* < 0.0001, *F*(3,32) = 28.70, one-way ANOVA; intact hands: *p* < 0.0001, *F*(3,32) = 23.52). The classification accuracies using SMF were significantly superior to those of other tested features (affected hands: SMF vs. alpha band power, *p* < 0.0001, SMF vs. beta band power, *p* = 0.0001, SMF vs. high-gamma band power, *p* < 0.0001, post-hoc Tukey-Kramer test; intact hands: SMF vs. alpha band power, *p* = 0.0001, SMF vs. beta band power, *p* = 0.0263, SMF vs. high-gamma band power, *p* < 0.0001). These findings demonstrate that the SMFs were capable of extracting motor information about movement type and intention with significantly higher accuracy than other frequency features of MEG signals. Moreover, using the SMFs, information about hand movements was successfully extracted from MEG signals during the attempted movements of even the affected hands with accuracy comparable to that during the actual movements of intact hands.

### Timing of Onset Detection in Open-Loop Session

**Figure 5:**
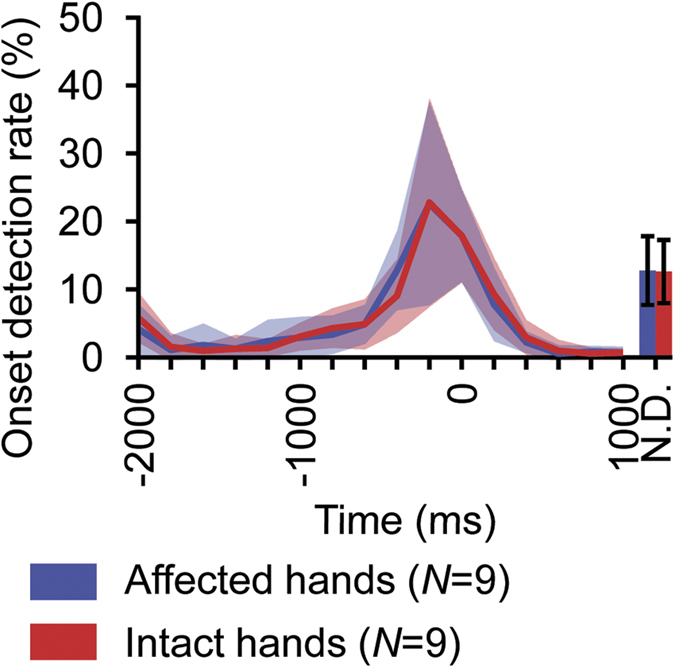
Offline evaluation of onset detection. Blue and red lines denote average of first onset detection rate in each time bin for movement of affected and intact hands, respectively. The shaded area shows standard deviation. The N.D. (not detected) bars denote rate of trials in which no onsets were detected. The error bars of the N.D. bars show their standard deviations. For each trial, first onset was searched beginning at −2000 ms. Time 0 ms denotes target time to detect, which is the training time of the class decoder in the training dataset.

To control the neuroprosthetic hand, we used an onset detection algorithm^14^ to infer the timing of movement intention using the SMFs (see Methods). The accuracy of the algorithm was evaluated for the MEG signals from −2000 to 1000 ms of the open-loop session (see Methods). The initial time of the inferred movement onset was evaluated in each trial by the nested cross-validation technique (see Methods). The algorithm successfully inferred the movement onset to be around the peak of the classification accuracy of movement type (0 ms, Fig. 5). Notably, the detected time of movement onset peaked at −200 ms for both affected and intact hands. Moreover, the onset was selectively inferred within ± 500 ms in 63.0 ± 15.6% (mean ± SD) and 63.1 ± 16.0% of trials, for affected and intact hands, respectively. Thus, the proposed algorithm succeeded in detecting the point in time at which the classification accuracy of the performed movement type was high.

### Online Control of the Prosthetic Hand

Finally, using the algorithm to detect the timing of movement intention and the trained decoder to infer movement types, five subjects were tested for their ability to control the prosthetic hand in a closed-loop condition (Fig. 1b). The prosthetic hand was controlled to form the inferred hand posture at the predicted onset of the attempted movement. The subjects successfully controlled the prosthetic hand by attempting to move their affected hands following the instructions to grasp with or open the prosthetic hand (see Methods, Fig. 1b). For example, subject 1 succeeded in achieving 10 correct inferred movement types out of 12 detected onsets within specific periods in which they were instructed to initiate movements (83.3%) and without any actual body movements (Supplementary Video 1). The accuracy to infer movement type was significant for two of the five subjects (Table 2; *p* < 0.05, one-tailed Fisher’s exact test). Moreover, the movement onset was selectively detected during the specific periods to initiate movements, with a statistical significance for two subjects (Table 3; *p* < 0.05, one-tailed Fisher’s exact test). Notably, to move the prosthesis into the instructed posture, the subjects needed, on average, only a few onset detections (1.28 ± 0.57 times, mean ± SD). Thus, we demonstrated that severely paralysed subjects successfully controlled the non-invasive neuroprosthetic hand in the closed-loop condition using attempted movement of their paralysed hands.

## Discussion

We previously developed and tested on healthy subjects a novel neuroprosthetic hand that used real-time MEG signals for movement^14^. Here, we tested this neuroprosthetic hand on severely paralysed patients and an amputee to evaluate their ability to control the prosthesis through the slow components of the MEG signals. We demonstrated that the slow components conveyed enough information about the affected hands to infer the timing and type of the attempted movements and to control a neuroprosthetic hand in real-time. Moreover, the slow components of the MEG signals appear to reflect the slow components of the cortical potentials in the sensorimotor cortex related to motor information. Although further studies are required, these results suggested that this MEG-based BMI might estimate the ability of paralysed patients to control an invasive neuroprosthesis using the slow cortical potentials of the ECoG signals.

Characteristic activations of the cortical potentials were observed in the sensorimotor cortex during the attempted movements of affected hands. Some invasive studies using ECoG or intracortical signals have reported that, in the sensorimotor cortex, the characteristic features of movement-specific activities such as evoked activity of alpha, beta and high-γ frequency ranges are preserved even in severely paralysed patients^2^,^6^,^31^. This is the first report in which a non-invasive whole brain study showed significant modulation of cortical currents depending on movement type. The characteristic time course of the SMF and the cortical distribution of the eSCP estimated from MEG signals suggest that the SMFs originated from the movement-related cortical potentials (MRCPs)^29^,^32^,^33^, which gradually increased prior to the movement onset, peaked during the movement, and were predominantly observed in the sensorimotor cortex contralateral to the movements. Our results show that even attempted movements of affected hands elicited significant MRCPs in the sensorimotor cortex contralateral to the hand and specifically depended on the attempted movement type. Actually, the requested movement type for the affected hand was inferred without significant difference in accuracy compared to that for the intact hand; however, even a small decrement in accuracy might be due to the lower amplitude of the MRCFs during the attempts to move the affected hand compared to that during actual movement.

In contrast to the similar activation in contralateral sensorimotor cortex, our results also showed that the ipsilateral sensorimotor cortex was differently activated during movements of intact and paralyzed hands. A previous study using transcranial magnetic stimulation suggested that there was cortical reorganization in the ipsilateral sensory motor cortex after amputation^26^. Our results are consistent with asymmetric brain activations for the intact and paralyzed hands. Notably, the current estimation technique from MEG signals demonstrated its potential for imaging brain activity related to the reorganization. Classification analysis using the estimated cortical currents would reveal cortical reorganization in the ipsilateral sensorimotor cortex in terms of neural information.

The MEG-based neuroprosthesis examined here is the first non-invasive BMI controlled by severely paralysed patients by combining motor information about both movement type and intention. Our previous study using ECoG revealed that motor information about movement intention was preserved even among severely paralysed patients, whereas motor information about movement type varied widely^6^. Most of the previously reported non-invasive BMIs controlled the prosthesis based on preserved motor information about movement intention extracted using alpha and beta frequency powers in the sensorimotor cortex^8^,^9^,^10^,^34^,^35^,^36^. However, as shown in our results, these features are not good at distinguishing multiple movement types and can only discriminate a moving (movement imaging) state from a resting state. By contrast, our MEG-controlled neuroprosthetic hand inferred not only movement timing, but also movement type. It enabled the paralysed patients more natural and sophisticated control of the prosthesis to perform multiple movements. Moreover, as the amount of motor information varies from patient to patient^6^, the proposed BMI can be adapted for individual patients based on how much motor information can be derived from their brain signals, thereby maximising performance by inferring movement timing in those patients without movement-type information.

The performance of our system showed comparable accuracy to recent MEG-based BMI study. It is difficult to compare our results to other studies, because our system is the first non-invasive BMI combining motor information about both movement type and intention. However, from the point of view of onset detection, Foldes *et al*. reported that paralysed patients succeeded to keep the BMI-controlled hand open for 5s in 28−64% of trials, with successful grasping in 63.3−76% of trials, using movement intention^37^. These accuracies are comparable to the performance in this study.

Moreover, the performance of our system might be improved, however, by refining the hardware and training the patients to use the prosthetic hand. Previous studies reported that the delay of visual feedback deteriorated tracking performance^38^,^39^,^40^. In our system, an intention required a delay of approximately 830 ms to control the prosthesis. The performance of the real-time control might be increased by improving the processing speed of the system. In addition, the results of this study indicated that some frequency features also contain information about movements. By improving the speed, the system may be able to handle these features, whose calculations are time-consuming. Moreover, previous studies revealed that the performance of the BMI was improved by training with feedback^8^,^41^. By combining minimisation of the delay and training, the performance of the prosthetic hand control is expected to improve.

The performance of our MEG-based neuroprosthesis might reflect a patient’s ability to control an invasive BMI using the SCPs in the sensorimotor cortex. The source localization analysis showed that the observed SMFs had characteristics of the MRCFs and originated from the SCPs in the sensorimotor cortex, which are used in the ECoG-based BMI^6^,^42^. Moreover, previous studies revealed that appropriate feedback using a real-time BMI was able to enhance cortical function^43^,^44^ and to improve the BMI performance itself 8,36. By using signals common to both the invasive and the non-invasive BMI, the proposed non-invasive BMI might possibly be used to evaluate and train a patient’s individual ability to control an invasive BMI.

## Methods

### Subjects

Eight brachial plexus root avulsion patients (Subject 1–3, 5–9) and one amputee (Subject 4) (nine men; mean age 50.8 years, range 38–58 years) participated in this study. All subjects were right-handed. Their clinical profiles are listed in Table 1. All subjects were informed of the purpose and possible consequences of this study, and written informed consent was obtained. All experimental procedures were performed in accordance with protocols approved by the ethics committee of Osaka University Hospital (# 12107).

### Task

#### Open-loop session

Subjects were instructed to grasp with or open the intact hand or to attempt these movements with the affected hand once at the time of the execution cues given visually and aurally every 5.5s, 40 times for each movement type (Fig. 1a). To reduce motion artefacts, the subjects were instructed to perform the attempt or actual movement without moving any other body part. The type of movement to perform was presented visually with either the Japanese word for “grasp” or “open.” After the movement type instruction, four execution cues were given to the subject. The order of the movement type instructions was randomized.

#### Closed-loop session

Visual feedback was given to subjects with a screen fixed in front of them, showing a picture of the prosthetic hand in real-time and the instruction monitor (Fig. 1b). The instruction monitor displayed either the Japanese word for “grasp” or “open” alternately every 7s for a total of 22 instructions. Subjects were told to control the prosthetic hand by following the instruction (grasp or open), using the same attempts to move their affected hand as in the open-loop session.

### Experimental Procedure

Subjects participated in one open-loop session in which they were instructed to move their intact hand, and in another open-loop session to attempt movements of their affected hand. Five subjects (subjects 1–5) joined one closed-loop session after the open-loop session for the affected hand to perform the online control of a prosthetic hand. In the closed-loop sessions, a real-time decoder, which was trained with decoding features calculated from the previous open-loop session, controlled the prosthetic hand. To control the prosthetic hand, the subjects were instructed to perform the same movements performed in the open-loop sessions. In the beginning of each closed-loop session, the experimenter modulated the thresholds of the real-time decoder to detect movement intention. Then, the subjects controlled the prosthesis with a fixed threshold to evaluate the performance of the prosthesis. To avoid fatigue, each subject participated in only one closed-loop session for evaluation, and did not have enough time to be trained with the prosthetic hand control.

### Recording Method and Data Collection

Figure 1b shows an overall schematic of the system. The subjects lay in a supine position with a cushion placed under their elbow to reduce artefacts caused by shoulder movements. A projection screen fixed in front of their face presented visual stimuli using a presentation system and a liquid crystal projector. Neuromagnetic brain activity was measured by a 160-channel whole-head MEG housed in a magnetically shielded room. The MEG signals were sampled at 1000 Hz with an online low-pass filter at 200 Hz and acquired online by FPGA DAQ boards after passing through an optical isolation circuit. Subjects were instructed not to move their head to avoid motion artefacts. The head position was measured by five marker coils attached to the subject’s face to estimate cortical currents before each session.

### Real-Time Decoding and Prosthetic Hand Control

Using an algorithm we developed previously^14^, a prosthetic hand was controlled by neuromagnetic activity evoked by the patient’s attempts to move their affected hands. MATLAB R2013a (Mathworks, Natwick, MA, USA) was used to calculate decoding features and to control the prosthetic hand in real-time. In the open-loop session, MEG signals from 84 parietal sensors (Fig. 1b) were averaged over 500 ms and were *z*-scored using the mean and standard deviations estimated from the initial 50s of the session to acquire the SMF. The SMF was calculated with its time window beginning at −2000 and continuing to 1000 ms with respect to the time of the execution cue, shifting by 100 ms. To control the prosthesis in the subsequent closed-loop sessions, a real-time decoder was trained using the SMFs from the previous open-loop session. In the closed-loop session, the real-time decoder estimated the confidence values of movement intention via the radial basis function (RBF) kernel SVM in LIBSVM toolbox^45^ and the Gaussian process regression^46^ in the GPML toolbox^47^, using the latest SMF every 200 ms (see Supplementary Fig. S1). We used an onset detection algorithm combining these two confidence values to detect the timing of the patient’s intention to move their affected hands. In the algorithm, an onset was detected when both of the confidence values exceeded their respective thresholds, which were set manually. To avoid multiple detections in a single intention, onsets detected within 1.5s from the first detection were ignored. At the time of the detected onset, the attempted movement type (grasp or open) was inferred by another RBF kernel SVM, and the prosthesis was controlled to form the hand shape of the inferred movement type. The prosthetic hand used in this study was developed by Dr. Hiroshi Yokoi to imitate the human distal upper limb. Ten servo motors controlled the joints in each finger, which had 2 degrees of freedom, using flexible wires in a coordinated manner to form a grasping or opening hand shape. The overall delay from the MEG system to the visual feedback of the prosthetic hand was around 830 ms in total: real-time data acquisition, ~20 μs; data processing, ~70 ms; the time window for the SMF, 500 ms; prosthetic hand control, ~150 ms; projection to screen in MEG room including video recording, ~110 ms.

### Analysis of Offline Data

#### Cortical current estimation by VBMEG

A reconstruction of the cortical surface was constructed based on MR structural images using FreeSurfer image analysis^48^. Using the VBMEG, we estimated 4004 single-current dipoles that were equidistantly distributed on and perpendicular to the cortical surface. The method calculated an inverse filter to estimate the cortical current for each dipole from the selected MEG sensor signals. The hyperparameters m_0_ and γ_0_ were set to 100 and 10, respectively. The inverse filter was estimated using MEG signals from 0 to 1000 ms relative to the execution cue, with a baseline of the current variance estimated from the signals from −1500 to −500 ms. The filter was then applied to sensor signals in each trial to calculate cortical currents.

#### Classification accuracy in the open-loop session

MEG signals from 84 parietal sensors were converted to SMF by averaging over 500 ms and normalizing to a *z*-score from a 50-s period at the beginning of the session in the same way as the online-acquired features. The powers of three frequency bands (alpha: 8−13 Hz; beta: 13−30 Hz; high-γ: 80−150 Hz) were calculated by the fast Fourier transform from the same signal used to obtain the SMF, and likewise converted to frequency-domain features. Classification accuracy to infer movement type was estimated with decoding features that were calculated at each 500-ms time window starting at −500 and continuing to 500 ms relative to the execution cue, shifting by 100 ms. Nested cross-validation was adopted for classification to avoid overestimation of the decoding accuracy^49^. Each test dataset was classified by a decoder trained with the RBF kernel SVM using decoding features at a certain time window and the hyperparameters gamma and cost of the SVM (see Supplementary Fig. S2). The time window and hyperparameters were selected only based on the training dataset; thus, they were always selected independently from the test dataset.

The movement intention was also inferred by nested cross-validation, optimizing the hyperparameters with combined decoding features of 11 time windows from −2500 to −1000 ms as resting and those from −500 to 1000 ms as features during intention. All decoding analyses were performed in MATLAB R2007b using the RBF kernel SVM.

#### Offline evaluation of onset detection

The algorithm of the real-time decoder was adapted to the SMFs in the open-loop session using ten-fold cross-validation to evaluate timing of the onset detection. The real-time decoder was trained with a training dataset, and the time of the first onset detection was pinpointed in each trial within the test dataset. The search started at −2000 ms relative to the time at which the classification accuracy of the movement types peaked within the training dataset, and continued until 1000 ms. During the searched period, the SMFs were tested at an interval of 200 ms.

### Analysis of Online Performance

To evaluate the selectivity of onset detection in the closed-loop session, the session was divided into two sections depending on the instruction relative to the current state of the prosthetic hand: “same-state” in which instruction and current state were the same (no need to move the prosthetic hand) and “different-state” in which they differed (need to move the prosthetic hand). Each section was classified by the detection of onset within the section (true positive: different-state section with onset detection; false positive: same-state section with onset detection; false negative: different-state section without onset detection; true negative: same-state section without onset detection; for an illustration of this evaluation method, see Supplementary Fig. S3). The recorded times of onset detections were corrected by subtracting 70 ms, the system delay required to detect onset. Selectivity of the onset detection in the closed-loop session was tested using a one-tailed Fisher’s exact test based on the classified sections. The decoding performance of movement types in the closed-loop session was evaluated by the number of correctly inferred movement types at all onset detections during which the patients intended to move their affected hands (different-state sections). Selectivity of the movement types in the closed-loop session was tested using a one-tailed Fisher’s exact test.

## Additional Information

**How to cite this article**: Fukuma, R. *et al*. Real-Time Control of a Neuroprosthetic Hand by Magnetoencephalographic Signals from Paralysed Patients. *Sci. Rep.* **6**, 21781; doi: 10.1038/srep21781 (2016).

## Supplementary Material

Supplementary Information

Supplementary Video 1

## Figures and Tables

**Table 1:** Clinical profiles of participants.

Subject ID	Age (y)/Sex	Diagnosis	Hand MMT [0–5]	Hand sensation	Disease duration (y)
1	58/M	R Avulsion	0	0	40
2	49/M	R Avulsion	0	0	29
3	50/M	R Avulsion	1	0	34
4	48/M	R Amputation	–	–	1.5
5	51/M	L Avulsion	0	0	6
6	56/M	R Avulsion	0	0	10
7	51/M	R Avulsion	0	0	11
8	56/M	L Avulsion	0	0	13
9	38/M	R Avulsion	0	0	20

R, L: right, left; Avulsion: brachial plexus root avulsion; MMT: manual muscle test.

**Table 2:** Summary of movement type decoding results in closed-loop prosthetic hand control.

Subject ID	Accuracy (%)	Movement instruction / Inferred movement				*p*-value
		Grasp/Grasp	Grasp/Open	Open/Grasp	Open/Open	
1	83.3	4	1	1	6	0.046
2	66.7	8	1	8	10	0.033
3	42.9	1	2	6	5	0.904
4	42.9	4	2	6	2	0.825
5	42.3	4	11	4	7	0.831

*p*-values are of one-sided Fisher’s exact test.

**Table 3:** Summary of movement onset detection results in closed-loop prosthetic hand control.

Subject ID	Accuracy (%)	TP	FP	FN	TN	*p*-value
1	58.3	11	7	8	10	0.253
2	64.0	21	14	4	11	0.031
3	74.2	13	6	2	10	0.006
4	63.6	12	7	5	9	0.114
5	62.5	17	12	3	8	0.078

*p*-values are of one-sided Fisher’s exact test; TP, FP, FN, TN: numbers of – true positive, false positive, false negative, true negative – detections.
