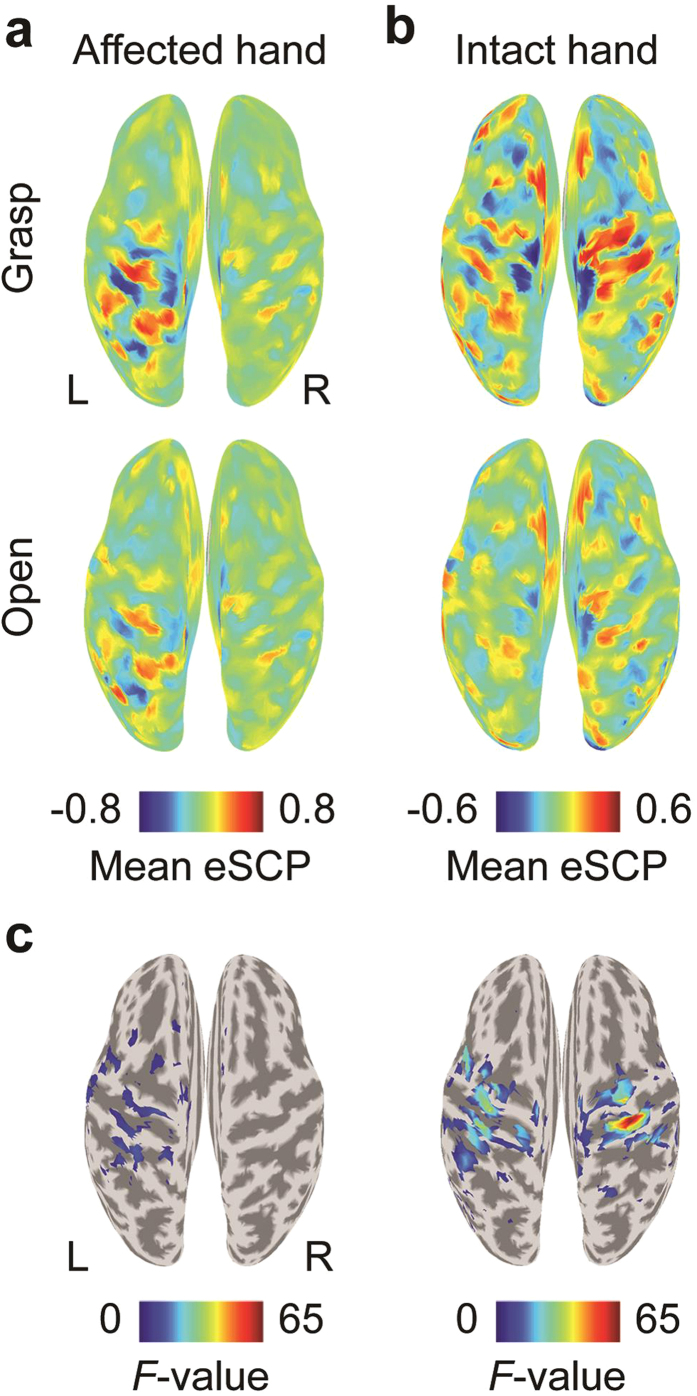# Corrigendum: Real-Time Control of a Neuroprosthetic Hand by Magnetoencephalographic Signals from Paralysed Patients

**DOI:** 10.1038/srep34970

**Published:** 2016-10-19

**Authors:** Ryohei Fukuma, Takufumi Yanagisawa, Youichi Saitoh, Koichi Hosomi, Haruhiko Kishima, Takeshi Shimizu, Hisato Sugata, Hiroshi Yokoi, Masayuki Hirata, Yukiyasu Kamitani, Toshiki Yoshimine

Scientific Reports 6: Article number: 2178110.1038/srep21781; published online: 02 24 2016; updated: 10 19 2016

This Article contains errors.

In Table 1, the heading ‘Hand MMT [0–5]’ should read ‘MMT [0–5]’. In addition, the disease durations of subjects 6 and 9 were incorrectly given as ‘10’ and ‘20’ years, respectively. These should read ‘38’ and ‘21’.

In Figure 3c, the upper limit of the colour bar is incorrectly given as ‘20’, and should read ‘65’. The figure legend is correct. The correct Figure 3 appears below as Figure 1.

## Figures and Tables

**Figure 1:**