# Supplementary material for: Real-Time Control of a Neuroprosthetic Hand by Magnetoencephalographic Signals from Paralysed Patients
Source: Sci Rep. 2016 Feb 24;6:21781. doi: 10.1038/srep21781 (PMC4764841; doi:10.1038/srep21781)
Supplement: Supplementary Information [file srep21781-s1.pdf]

Supplementary Information

Real-Time Control of a Neuroprosthetic Hand by Magnetoencephalographic Signals from Paralysed Patients

Ryohei Fukuma[1,2,3,+], Takufumi Yanagisawa[1,2,4,*,+], Youichi Saitoh[1,5], Koichi Hosomi[1,5], Haruhiko Kishima[1], Takeshi Shimizu[1,5], Hisato Sugata[1], Hiroshi Yokoi[6], Masayuki Hirata[1], Yukiyasu Kamitani[2,3,7], Toshiki Yoshimine[1]

[1] Osaka University Graduate School of Medicine, Department of Neurosurgery, Suita 565-0871, Japan

[2] ATR Computational Neuroscience Laboratories, Department of Neuroinformatics, Seika-cho 619-0288, Japan

[3] Nara Institute of Science and Technology, Graduate School of Information Science, Ikoma 630-0192, Japan

[4] Osaka University Graduate School of Medicine, Division of Functional Diagnostic Science, Suita 565-0871, Japan

[5] Osaka University Graduate School of Medicine, Department of Neuromodulation and Neurosurgery, Suita 565-0871, Japan

[6] The University of Electro-Communications, Department of Mechanical Engineering and Intelligent Systems, Chofu 182-8585, Japan

[7] Kyoto University, Graduate School of Informatics, Kyoto 606-8501, Japan

* tyanagisawa@nsurg.med.osaka-u.ac.jp

[+] These authors contributed equally to this work

Supplementary Video 1. Real-time control of a neuroprosthetic hand by paralysed patient. Video shows the real-time control of the prosthetic hand by subject 1 using attempts to grasp with or open his completely paralysed right hand.

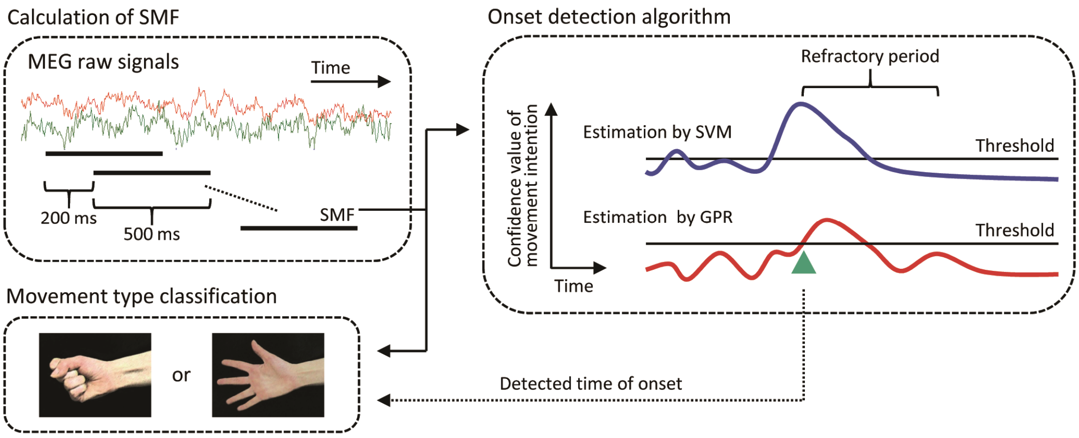

Supplementary Figure S1. Online decoding process. MEG signals of 500-ms time windows were converted to SMF for every 200 ms. Using the acquired SMFs, an onset detection algorithm estimated the times of movement intentions. In the algorithm, confidence values of movement intention were calculated by a support vector machine (SVM) and a Gaussian process regression (GPR). When both of these values exceeded their respective, manually set thresholds, onset was detected (indicated by green arrowhead). To avoid multiple detections of onset in a single intention, onsets within a refractory period were ignored. At the time of the detected onset, movement type was classified by another decoder trained by the SVM.

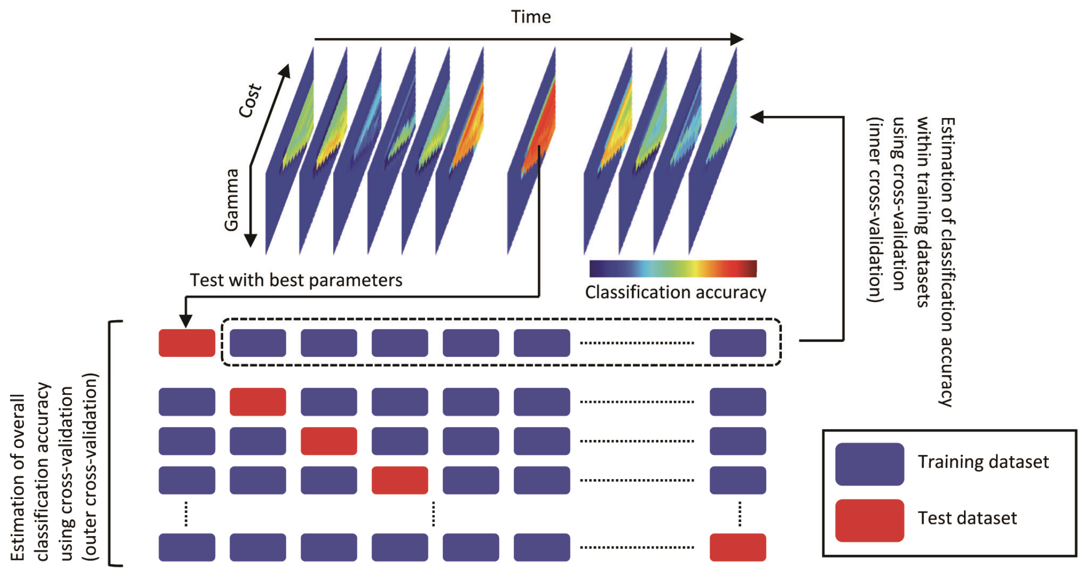

Supplementary Figure S2. Classification analysis using nested cross-validation. To assess the classification accuracy without overestimation, nested cross-validation was adopted. For each round of outer cross-validation, training datasets were evaluated for classification accuracy using cross-validation (inner cross-validation). The inner cross-validation was performed repeatedly at each time window of the decoding feature and with all combinations of gamma and cost, the hyperparameters of the RBF kernel SVM. The time window and hyperparameters that maximised the classification accuracies were selected as optimal parameters. Finally, the test dataset of the outer cross-validation was tested by a decoder trained with the optimal hyperparameters using decoding features at the optimal time window.

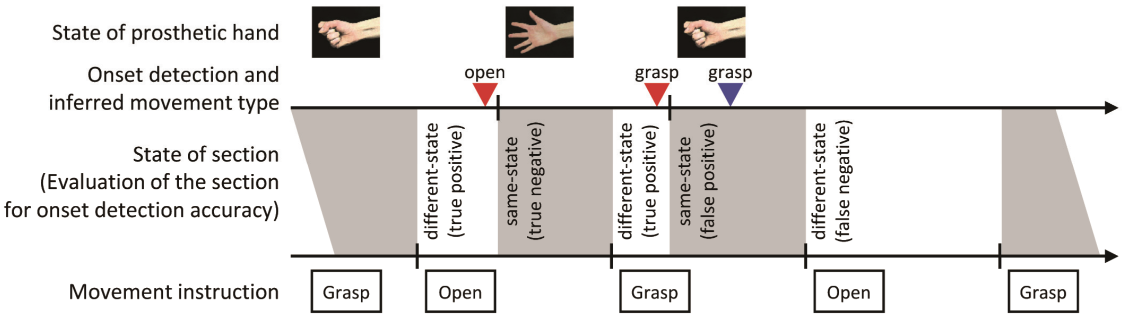

Supplementary Figure S3. Evaluation method of closed-loop sessions. Posture of hand denotes the state of the prosthetic hand. Grey and white areas between arrows denote "same-state" and "different-state" sections, respectively. These sections were defined by the movement instructions relative to the state of the prosthetic hand. Arrowheads with "grasp" or "open" indicate the timing of onset detections and inferred movement at the detection. The onsets indicated by red arrowheads were evaluated for their movement type classification accuracy in the closed-loop session, whereas the onset shown with the blue arrowhead was not used. At the blue arrowhead, we expected that the patient had no intention of moving their hands. Or, at that time, we did not expect a neural signal corresponding to a movement intension. Therefore, we excluded the data of this onset from the evaluation of the movement type classification accuracy, because we could not evaluate *p*(movement type | neural signal for movement intention). It should be noted that the duration between onset detection and change in the state of the prosthetic hand corresponds to the system delay.
